# Establishing normative foot posture index values for the paediatric population: a cross-sectional study

**DOI:** 10.1186/s13047-016-0156-3

**Published:** 2016-07-26

**Authors:** Gabriel Gijon-Nogueron, Jesus Montes-Alguacil, Pilar Alfageme-Garcia, Jose Antonio Cervera-Marin, Jose Miguel Morales-Asencio, Alfonso Martinez-Nova

**Affiliations:** 1 Department of Nursing and Podiatry, Arquitecto Francisco Peñalosa 3, Malaga, Spain; 2 Department of Nursing, University of Extremadura, Badajoz, Spain

## Abstract

**Background:**

The Foot Posture Index (FPI) is an observational tool designed to measure the position of the foot. Its reliability is well established, and it provides normative reference values for the general population. However, this is not so for the paediatric population. The aim of this study is to determine FPI reference values in childhood, taking into account age and gender.

**Methods:**

This cross-sectional study included 1,762 school children (863 boys and 899 girls) aged 6–11 years, from Málaga, Granada and Plasencia (Spain). In every case, FPI measurements were obtained for both feet by two experienced podiatrists. A descriptive analysis was then conducted and the percentiles of the variables determined, with a significance level of *P* < 0.05.

**Results:**

The consolidated FPI results for the sample population produced mean values of 3.74 (SD 2.93) points for the right foot and 3.83 (SD 2.92) for the left. The 50th percentile was 4 points for both genders and for both feet, except for the right foot among the girls, which was slightly lower, at 3 points. The 85th percentile, which is considered to represent the boundary between the normal and the pronated foot among children, was 6 points, uniformly among the subjects.

**Conclusions:**

As a normative FPI value for the paediatric population, we recommend the 50th percentile, i.e. 4 points, for children, of both genders, aged 6 years. This value progressively falls with age, to 3 FPI points for children aged 11 years. The 85th percentile for the pronated foot and the 4th percentile for the supinated foot can be considered the pathological boundary.

## Background

In recent decades, the question of paediatric asymptomatic and flexible flatfoot (PAFF) has generated much controversy within the scientific community, and the debate continues today [1–5]. It is clear that rigid flatfoot and symptomatic flexible flatfoot (PSFF) in children [6] require proper evaluation [7]. Nevertheless, it is generally agreed that, to a greater or lesser extent, the incidence of PAFF as a physiological condition decreases as the child grows older [8].

Various ways of measuring PAFF have been proposed, ranging from simple examination of the footprint [9–12] to radiographic measures of the angle of the medial longitudinal arch [13, 14] to observational tests such as that of the heel rise [15] or the navicular drop [16–18], although few of these have been validated for use with children. As a result, clinicians are forced to make diagnostic decisions based on their personal experience with cases of PAFF [2].

The validation of foot measurement tests for paediatric patients would reduce the risk of errors being made in profiles of clinical normality, and could complement traditional diagnostics methods such as radiology. These tests could even serve as an initial screening method in large-scale analyses, prior to the application of more complex and/or specific tests.

The Foot Posture Index (FPI) is an observational measurement instrument that takes into account the three-dimensional nature of foot posture and has been shown to achieve good reliability in adults [19, 20] and in children [19, 21–23]. It has also been considered an appropriate measure for subjects who are not in good health [24–27]. Reference FPI values have been established for the adult population [20], but during childhood foot posture changes with growth and, to the best of our knowledge, no FPI reference values have been proposed for this population.

In view of the above considerations, the contribution of this study is that we establish normative values for age-related FPI in the paediatric population, with respect to the age group that is most susceptible to changes in foot posture.

## Methods

### Participants

This cross-sectional study involved 1,762 school children (863 boys and 899 girls) aged 6–11 years. Measurements were taken during 2013 and 2014, at ten schools randomly selected from 25 located in the provinces of Málaga, Granada and Plasencia (Spain). The average age of the sample was 8.29 years (SD 1.72) and the mean BMI of the children was 18.94 (SD 3.65 kg/m^2^) in the boys and 18.89 (SD 3.64 kg/m^2^) in the girls. The difference between genders was not statistically significant (*p* = 0.834) (Table 1).

**Table 1:** Descriptive for age, weight, height and BMI of the entire sample by gender

		Total				Male				Female		
	Mean	95 % CI		SD	Mean	95 % CI		SD	Mean	95 % CI		SD
BMI (Kg/m2)	18.87	18.70	19.04	3.62	18.91	18.67	19.14	3.64	18.83	18.59	19.07	3.62
Age (year)	8.17	8.09	8.24	1.60	8.12	8.01	8.22	1.57	8.21	8.11	8.22	1.62
Weight (Kg)	32.70	32.28	33.12	8.97	32.70	32.08	33.31	9.16	32.71	32.13	33.28	8.78
Height( Metre)	1.31	1.30	1.32	0.12	1.31	1.30	1.32	0.12	1.31	1.30	1.32	0.11

The inclusion criterion was that the children should be 6–11 years old. The exclusion criteria included the presence of pain in the foot at the time of physical examination, injury to the lower limbs, such as musculoskeletal injuries, during the previous 6 months, congenital structural abnormalities, cerebral palsy, motor dysfunction or prior surgery affecting the foot. The parents were previously informed about the characteristics of the study. They were all asked to complete a questionnaire and to provide signed consent to confirm the participation of their children in the study. This study was conducted in accordance with the Declaration of Helsinki and was approved by the Ethics Committees of the Universities of Extremadura and Málaga (Spain).

### Procedure

Foot posture was assessed by measuring the FPI with the subjects barefoot, in a relaxed standing position on a bench at 50 cm above the floor to facilitate visual and manual inspection. The FPI consists of the following six items referring to the position of the forefoot, midfoot and hindfoot, and the three planes of motion: 1) talar head palpation; 2) symmetry of supra and infra lateral malleolar curvature; 3) inversion/eversion of the calcaneus; 4) prominence in the region of the talus-scaphoid joint; 5) height of the medial longitudinal arch; 6) abduction/adduction of the forefoot. The FPI thus obtained ranges from -12 (highly supinated) to +12 (highly pronated) [19]. Inter-observer reliability for the FPI in the paediatric population is reflected in the consistent weighted Kappa value obtained (Kw = 0.86) by Morrison & Ferrari [22] in a sample of children aged 5–16 years. In our study, the FPI values were measured by two podiatrists (JMA and PAG) who are experienced in the use of this instrument. Both researchers measured the same 30 children and the inter-correlation coefficient (ICC) was calculated by reference to the same sample. Both podiatrists were blinded by using a folding screen, which was placed between the subject and the assessor, and only the foot and 10 cm of shank were visible. Participants were assessed while in a relaxed standing position, on a bench 50 cm tall to enable visual and manual inspection. Good inter-observer reliability was recorded (I.C.C. 0.852–0.895).

In all other respects of measurement, the protocol described by Redmond et al. in their manual for the FPI [28] was used.

### Data analysis

Statistical analysis was performed with SPSS.22 Software (SPSS Inc., USA). After checking the normality of the distribution (Kolmogorov-Smirnov) and the homogeneity of the data (Levene) in both samples, descriptive statistics were used to characterise the sample. The FPI was also analysed as continuous data rather than as z-score data [19]. A descriptive and frequency analysis of the variables was conducted, and the means, standard deviation and percentiles were determined. Student’s T-test was used as inferential proof for related samples (FPI, gender and age). The level of statistical significance was set at *p* < 0.05.

## Results and discussion

For the whole sample, the mean FPI obtained was 3.77 points (SD 2.93) for the right foot and 3.87 points (SD 2.92) for the left foot. By gender, the average values for the right foot were slightly higher in the boys than in the girls, with values of 3.93 (SD 2.99) versus 3.61 (SD 2.86), respectively, and the difference was statistically significant (*p* = 0.026). The mean values for the left foot were 4.00 (SD 2.96) in the boys and 3.74 (SD 2.87) in the girls (Table 2).

**Table 2:** Descriptions for the FPI of the whole sample. Detailed separate items, laterality and gender

	Gender														
	Total					Male					Female				
	Mean	95 % CI		Median	SD	Mean	95 % CI		Median	SD	Mean	95 % CI		Median	SD
FPI talar head palpation R	0,9	0,88	0,93	1	0,619	0,92	0,88	0,97	1	0,622	0,85	0,93	1	0,616	0,85
FPI curvature at the lateral malleoli R	0,57	0,54	0,6	1	0,63	0,6	0,56	0,64	1	0,633	0,49	0,58	0	0,626	0,49
FPI inversion/eversion of the calcaneus R	0,56	0,53	0,59	1	0,635	0,59	0,54	0,63	1	0,655	0,5	0,58	1	0,615	0,5
FPI talonavicular bulging R	0,53	0,5	0,56	0	0,647	0,57	0,52	0,61	0	0,661	0,46	0,55	0	0,632	0,46
FPI congruence of the medial longitudinal arch R	0,54	0,51	0,58	1	0,649	0,58	0,53	0,62	1	0,67	0,47	0,55	1	0,627	0,47
FPI abduction/adduction of the forefoot on the rearfoot R	0,62	0,59	0,65	1	0,668	0,65	0,61	0,7	1	0,678	0,55	0,64	1	0,657	0,55
FPI R score total	3,73	3,6	3,87	3	2,938	3,91	3,71	4,11	4	3,011	3,37	3,75	3	2,857	3,37
FPI talar head palpation L	0,94	0,91	0,97	1	0,606	0,96	0,92	1	1	0,614	0,88	0,96	1	0,598	0,88
FPI curvature at the lateral malleoli L	0,57	0,54	0,6	1	0,631	0,6	0,56	0,65	1	0,632	0,49	0,57	0	0,628	0,49
FPI inversion/eversion of the calcaneus L	0,59	0,56	0,62	1	0,641	0,61	0,57	0,66	1	0,649	0,53	0,62	1	0,633	0,53
FPI talonavicular bulging L	0,54	0,51	0,57	0	0,641	0,58	0,53	0,62	1	0,651	0,46	0,55	0	0,629	0,46
FPI congruence of the medial longitudinal arch L	0,55	0,52	0,58	1	0,651	0,59	0,54	0,63	1	0,673	0,48	0,56	1	0,629	0,48
FPI abduction/adduction of the forefoot on the rearfoot L	0,64	0,61	0,67	1	0,651	0,67	0,63	0,71	1	0,664	0,57	0,65	1	0,638	0,57
FPI L score total	3,83	3,69	3,97	4	2,932	3,98	3,78	4,18	4	2,987	3,5	3,87	3	2,873	3,5

By age groups, statistically significant differences between the genders (*p* < 0.05) were only observed in the children aged 6 years (right foot) and 7–12 years (left foot) (Table 3).

**Table 3:** Total FPI scores by age and laterality

		N		Mean (SD)		*P* value
Age	Lateral	Male	Female	Male	Female	
6	Right	128	126	4.8(2.9)	4.1(2.8)	<0.05
	Left	128	126	5.0(2.9)	4.2(2.7)	0.068
7	Right	169	188	4.3(2.9)	3.7(3.0)	0.086
	Left	169	188	4.4(2.8)	3.7(3.1)	<0.05
8	Right	196	166	4.1(3.1)	3.8(2.7)	0.383
	Left	196	166	4.0(3.0)	3.9(2.8)	0.523
9	Right	128	135	3.3(2.9)	3.2(2.8)	0.792
	Left	128	135	3.3(2.8)	3.4(2.8)	0.728
10	Right	113	143	3.2(2.8)	3.4(2.8)	0.515
	Left	113	143	3.3(2.8)	3.6(2.9)	0.301
11	Right	126	140	3.5(3.1)	3.1(2,8)	0.214
	Left	126	140	3.7(3.2)	3.2(2.7)	0.214

**Fig. 1:**
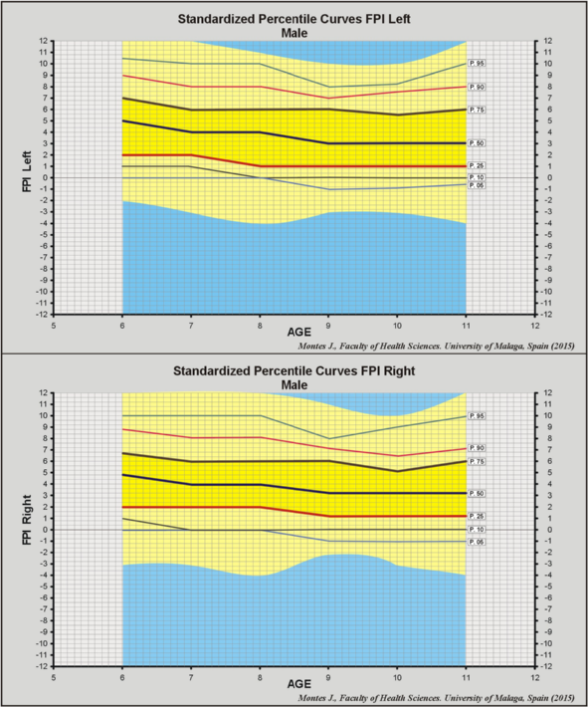
Percentile curves left and right foot in males

**Fig. 2:**
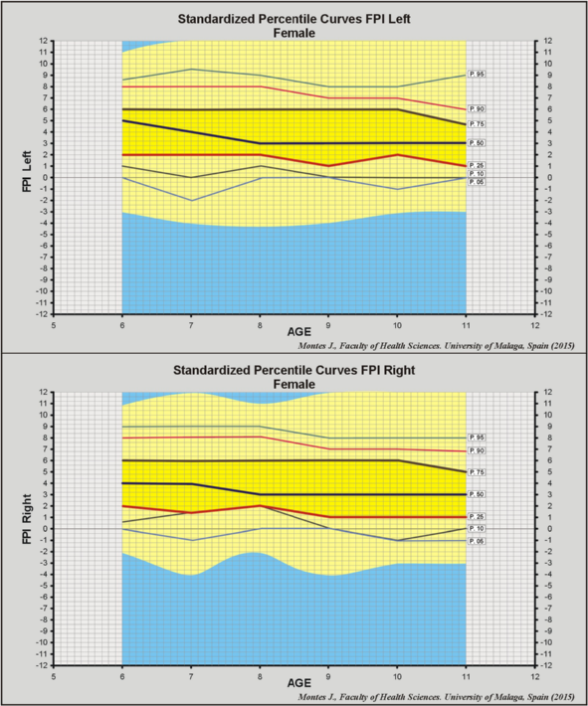
Percentile curves left and right foot in females

The FPI values were distributed into percentiles, as a continuous variable. A mean value of 4 points was obtained for the 50th percentile in both genders and for both feet, except the right foot in the girls, for which the value was slightly lower, at 3 points. The 75th sample produced uniform results, with 6 points in every case, and this value is considered the dividing line between physiological and pathological pronation. The mean FPI values and percentiles obtained for children aged 6–11 years are shown in four graphs, one for each gender and foot (see Figs. 1 and 2). In these graphs, the 50th percentiles are represented by the thick black line, the 25th percentiles by the thick red line and the 75th percentiles by the thick brown line, while thin lines represent the 90th (pink), 95th (dark blue), 10th (dark grey) and 5th (light blue) percentiles.

**Fig. 3:**
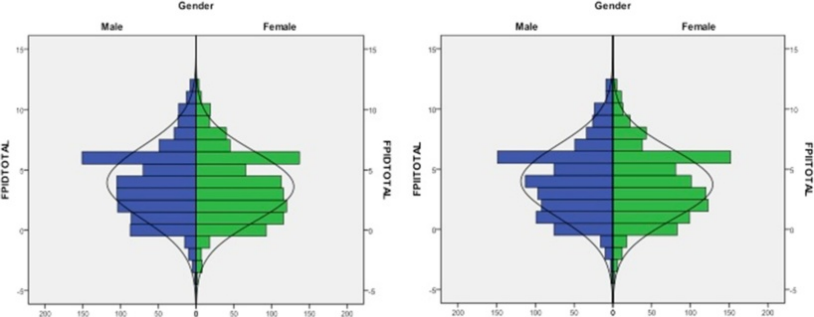
FPI frequencies in both sides and genders

As can be seen in the frequency graphs, FPI = 6 is the normal value for both genders and for both feet in the total sample (Fig. 3).

**Fig. 4:**
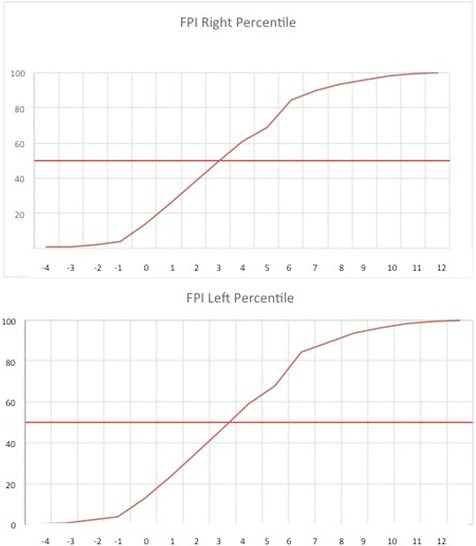
Percentile curves left and right foot with z-scores

In addition, the FPI of the z-score measurements was calculated. In this respect, the 50th percentile in both feet gave a FPI value of 4 (Table 4, Fig. 4). Following the criteria used by Redmond et al. [20], the 85th percentile was the boundary for the pronated foot, and the 4th percentile was that for the supinated foot.

**Table 4:** Total FPI with z-score by laterality

	FPI Right			FPI Left		
value	percentil	z	T	percentil	z	T
−4	1	−2.65	23.50	.2	−2.69	23.08
−3	1	−2.31	26.91	.7	−2.35	26.50
−2	2	−1.97	30.32	1.9	−2.01	29.92
−1	4	−1.63	33.73	3.8	−1.67	33.34
0	14	−1.29	37.14	12.6	−1.32	36.76
1	25	−0.94	40.55	23.5	−0.98	40.18
2	37	−0.60	43.96	35.4	−0.64	43.60
3	49	−0.26	47.37	47.3	−0.30	47.02
4	61	0.08	50.78	59.1	0.04	50.44
5	69	0.42	54.19	67.8	0.39	53.86
6	85	0.76	57.60	84.4	0.73	57.28
7	90	1.10	61.01	89.2	1.07	60.70
8	94	1.44	64.42	93.4	1.41	64.12
9	96	1.78	67.84	96.1	1.75	67.54
10	98	2.12	71.25	98.1	2.10	70.95
11	99	2.47	74.66	99.2	2.44	74.37
12	100	2.81	78.07	100	2.78	77.79

The aim of this study was to establish normal values for FPI in the paediatric population, in relation to foot posture changes with growth. No significant differences between genders were recorded for FPI frequencies and percentiles (Figs. 1 and 2). The study population was composed of 1,762 school children (863 boys and 899). This sample size and distribution is similar to that used in a study to establish normal values for FPI in the general population [20], which featured 1,648 participants (717 men, 825 women and 116 unspecified gender). The mean FPI value reported in this study (3.82 points) was also consistent with that for the children in our study (3.72 points). A noteworthy finding was that of the FPI values for younger and older adults (2.4 and 2.9 points, respectively), which highlights the existence of a significant difference in foot posture between children and adults (*F* = 51.07, *p* < 0.001) [20].

By gender, the mean FPI values were 3.96 and 3.67 points in boys and girls, respectively, which is within the generally-accepted margins of normal foot posture (0 to + 5 points) [20, 22], and coincides with the results obtained in previous studies of FPI in children [22, 29]. The value of the 85th percentile of the FPI was approximately 6 points for both genders (Table 4). Pronation is generally assumed to be represented by FPI values of 6–9 points for moderate pronation, and of 9–12 points for severe pronation [22], and therefore the 85th percentile can be taken as a cutoff point between normal and pronation.

One of the most interesting findings of this study is the small degree of change between the ages of 6 and 8 years. In fact, a significant reduction in PAFF only occurred from the age of 9 years. This outcome may be associated with physical development, which would corroborate previous studies that have reported a reduction in the prevalence of PAFF with age, despite using other measurement techniques such as examination of the footprint, radiographic measurement of angles or observational techniques [8, 13, 23–25, 30]. Our own study was based on observation of a large, multicentre and homogeneous sample, although restricted to a single country.

The main limitation of this study concerns the age of the subjects, who were only analysed up to age 11 years, a cutoff point determined by the fact that in Spain children transfer from primary school to high school at this age. Ideally, our sample should have included subjects aged up to 18 years.

Future studies of this type, with similarly large sample sizes and a greater age range, taking into account different ethnic groups and geographic regions, could achieve greater precision regarding the reference values for foot posture in childhood and adolescence. Moreover, cohort studies of foot posture in childhood, using the FPI, would contribute to a better understanding of the development of the foot during growth.

Our study shows the FPI to be a good outcome measure for foot posture in the paediatric population, achieving a weighted Kappa value of Kw = 0.88 [22], good intra-observer correlation (ICC = 0.93–0.96) and good inter-observer reliability (ICC = 0.79) [26]. This index has also been used successfully in studies based on subjects with diverse musculoskeletal pathologies [27, 31–33] and in studies of childhood obesity and its influence on foot posture [29, 34]. Furthermore, the FPI offers a three-dimensional assessment of pronation and supination, unlike traditional biplanar techniques such as footprint examination or radiography.

The results obtained in this study enable us to establish reference foot posture values, based on the FPI, for a healthy paediatric population. The data are presented by percentiles, with mean values and the corresponding standard deviation, which we believe would be useful in future studies conducted to identify non-normal values, whether physiological or clinical, identified in foot-screening procedures similar to those used to determine the BMI. This approach could be of value in identifying subjects who need to be referred elsewhere for deeper analysis of gait or growth. These data can also be used, in conjunction with those provided by future studies of foot symptomatology, to better determine what should be considered physiological or pathological as regards foot posture in childhood. Our study, therefore, could be viewed as complementary to Redmond et al. [20], regarding normative values for the Foot Posture Index in children.

## Conclusions

As limits for normative FPI values, we recommend the 85th percentile for pronation and the 4th for supination. The 50th percentile is 4 points for children (both male and female) aged 6 years. This value falls progressively with age, to 3 FPI points for children aged 11 years.

## Abbreviations

FPI, foot posture index; ICC, inter-correlation coefficient; PSFF, flatfoot and symptomatic flexible flatfoot
